# Probiotic supplementation for anxiety symptoms in people with Parkinson’s disease: a randomized, double-blind, placebo-controlled trial

**DOI:** 10.1038/s41531-026-01364-1

**Published:** 2026-04-25

**Authors:** Joyce S. T. Lam, Kira N. Tosefsky, Nicholas J. Ainsworth, Dylan Meng, Petra Uzelman, Julie Zhu, Mihai S. Cirstea, Fabricio Pio, Fidel Vila-Rodriguez, Andrew K. Howard, B. Brett Finlay, Silke Appel-Cresswell

**Affiliations:** 1 Pacific Parkinson’s Research Centre, Djavad Mowafaghian Centre for Brain Health, University of British Columbia, Vancouver, Canada; 2 MD/PhD Program, Faculty of Medicine, University of British Columbia, Vancouver, Canada; 3 Non-Invasive Neurostimulation Therapies Laboratory, Department of Psychiatry, University of British Columbia, Vancouver, Canada; 4 Department of Psychiatry, University of British Columbia, Vancouver, Canada; 5 Michael Smith Laboratories, University of British Columbia, Vancouver, Canada; 6 Department of Microbiology and Immunology, University of British Columbia, Vancouver, Canada; 7 School of Biomedical Engineering, University of British Columbia, Vancouver, Canada; 8 Department of Biochemistry and Molecular Biology, University of British Columbia, Vancouver, Canada; 9 Division of Neurology, Department of Medicine, University of British Columbia, Vancouver, Canada

**Keywords:** Diseases, Gastroenterology, Health care, Medical research, Microbiology, Neuroscience

## Abstract

Anxiety is a prevalent non-motor symptom of Parkinson’s disease (PD), yet treatment options remain limited. This randomized, double-blind, placebo-controlled trial evaluated the effects of a 12-week probiotic supplement containing nine bacterial strains (*Bifidobacterium bifidum* W23, *Bifidobacterium lactis* W51 and W52, *Lactobacillus acidophilus* W37, *Levilactobacillus brevis* W63, *Lacticaseibacillus casei* W56, *Ligilactobacillus salivarius* W24, and *Lactococcus lactis* W19 and W58) on anxiety symptoms in 61 individuals with PD and clinically significant anxiety. Both the probiotic (*n* = 30) and placebo (*n* = 31) groups showed significant within-group improvements on the Parkinson Anxiety Scale, with no significant between-group differences. The probiotic group showed a statistically significant improvement on the Montreal Cognitive Assessment compared with placebo (adjusted mean difference of 1.1 points; 95% confidence interval: 0.04–2.1; *p* = 0.043). No treatment effects were seen on other secondary outcomes, including depression, constipation, or motor symptoms. No significant between-group differences in gut microbiota composition or systemic inflammatory markers were observed. While the multi-strain probiotic did not reduce anxiety more than placebo, its potential cognitive effects warrant further investigation in larger trials. This trial was registered on ClinicalTrials.gov (NCT03968133) on May 28, 2019.

## Introduction

In addition to its hallmark motor symptoms, Parkinson’s disease (PD) is associated with a wide range of non-motor features. Anxiety is among the most frequently reported, with clinically significant symptoms present in ~25–30% of people with PD (PwP)^1,2^. Anxiety in PD is linked to worsening motor, cognitive, and functional performance^3–5^, making it a major determinant of health-related quality of life^6,7^. However, it remains under-recognized and under-treated in PwP^8,9^, partly due to its phenomenological overlap with other non-motor symptoms such as depression and autonomic dysfunction^10^, and its frequent fluctuation alongside motor symptoms^11^.

Pharmacological management of anxiety in PD typically involves selective serotonin reuptake inhibitors (SSRIs) and benzodiazepines^9,12^. While both medication classes are useful in the appropriate setting, SSRIs can cause sleep disturbances, somnolence, and gastrointestinal issues^13^, and benzodiazepine use increases the risk of cognitive impairment and falls^12^. Evidence supporting these treatments in PD remains limited^14,15^. To date, only three randomized controlled trials (RCTs) have specifically targeted anxiety as a primary outcome in PwP with clinically significant anxiety. One pharmacological RCT assessing buspirone reported tolerability concerns and worsening of motor symptoms^16^. Two additional RCTs investigated non-pharmacological approaches—cognitive behavioral therapy (CBT)^17^ and acupuncture^18^. While both showed promise, limitations such as lack of blinding in CBT studies^14^ and variability in acupuncture delivery and cultural factors^18^ highlight the need for further well-controlled trials.

Perturbations of the gut microbiota have been implicated in the pathophysiology of both PD and anxiety, through potential mechanisms involving gut barrier dysfunction, immune dysregulation, and alterations in neurotransmitter levels^19,20^. These observations have led to growing interest in microbiota-targeted therapies, with more than 10 probiotic RCTs conducted for the management of various PD symptoms^21–32^ and over 20 RCTs for alleviating anxiety symptoms in non-PD populations to date^33,34^, many of which have reported beneficial effects. In PD, probiotics are well tolerated, with Class I evidence supporting their use for treating constipation^21,25^ and some preliminary evidence suggesting benefits for motor^24,27^ and depressive symptoms^27,28,30^. A few studies have also reported benefits for PD-related anxiety^27,28,30^, but the quality of evidence is limited, as anxiety was assessed as a secondary outcome using mostly non-PD-specific measures, and many participants had minimal or no significant anxiety^35^. Additionally, most trials lacked biological endpoints, making it challenging to determine underlying mechanisms. To address these outstanding questions, we conducted an RCT evaluating the effects of a 12-week probiotic intervention on anxiety symptoms in PwP.

## Results

### Participants

**Fig. 1:**
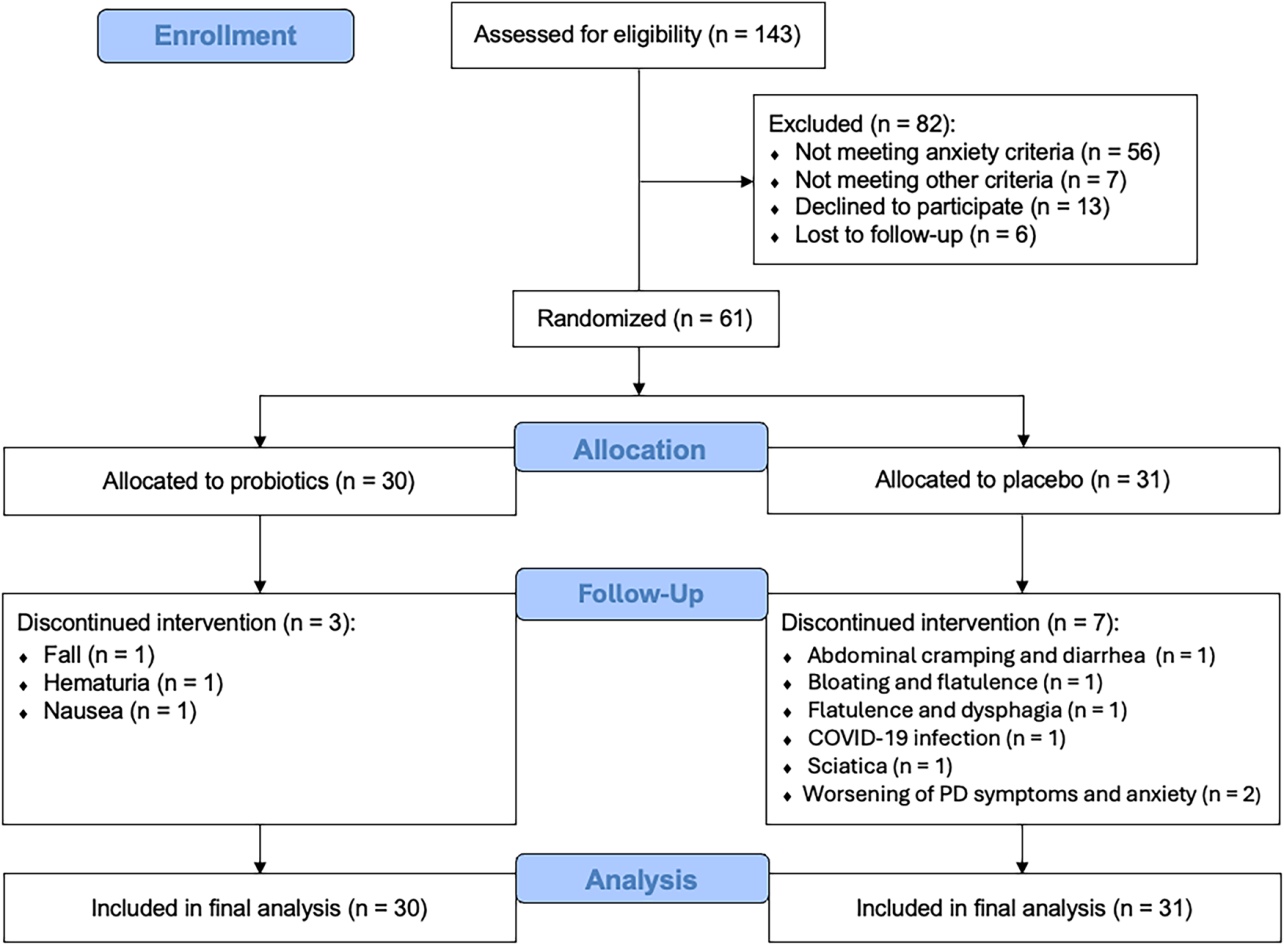
CONSORT flow diagram. Participant flow through screening, randomization, follow-up, and inclusion in the final intention-to-treat analysis.

A total of 143 individuals were screened between December 2020 and February 2023, with the final participant completing the study in July 2023. Participant flow, including exclusions and withdrawals, is reported in Fig. 1. Sixty-one PwP with anxiety were enrolled and randomly assigned to either the probiotic group (*n* = 30) or the placebo group (*n* = 31). Ten participants (16%) withdrew from the study after randomization: three in the probiotic and seven in the placebo groups. Reasons for discontinuation and their relationship to the intervention are provided in Supplementary Table 5. All 61 randomized participants were included in the intention-to-treat (ITT) analysis.

Participants were predominantly male (68.9%) and Caucasian (82.0%). The mean (standard deviation; SD) age of the study population was 64.2 (8.0) years, with a mean disease duration of 7.5 (4.9) years. Thirty-four participants met exactly one anxiety criterion, 19 fulfilled two criteria, and 8 satisfied all three (Supplementary Table 6). Fifty-four participants met the Parkinson Anxiety Scale (PAS) threshold. Baseline demographic and clinical characteristics were well balanced between the two groups (Table 1 and Supplementary Table 7). The mean PAS score was 18.1 (4.3) in the probiotic group and 18.5 (5.6) in the placebo group. No significant differences were found, except for a higher number of participants taking antidepressants in the placebo group (*p* = 0.040).

**Table 1:** Demographic and clinical characteristics at baseline

	Probiotic (*n* = 30)	Placebo (*n* = 31)	*p*^a^
Age, years	63.8 (7.9)	64.7 (8.3)	0.66
*Sex*, *n* *(%)*			0.79
Male	20 (66.7%)	22 (71.0%)	
Female	10 (33.3%)	9 (29.0%)	
Education, years	16.4 (3.3)	16.8 (2.7)	0.60
Currently in a relationship, *n* (%)	25 (83.3%)	29 (93.5%)	0.26
*Race/ethnicity*, *n* *(%)*			
Caucasian	25 (83.3%)	25 (80.6%)	1.0
Asian	4 (13.3%)	4 (12.9%)	1.0
First Nations	1 (3.3%)	0 (0%)	0.49
Latino/Hispanic	0 (0%)	1 (3.2%)	1.0
Other	0 (0%)	1 (3.2%)	1.0
Body mass index, kg/m^2^	26.8 (4.5)	25.1 (4.1)	0.12
PASIPD, MET h/day	21.6 (14.6)	16.8 (11.3)	0.16
*PD-related variables*			
Disease duration, years	8.1 (5.2)	6.9 (4.7)	0.36
LEDD^b^, mg	997.3 (483.9)	828.1 (455.1)	0.17
Levodopa use, *n* (%)	27 (90.0%)	30 (96.8%)	0.35
Hoehn and Yahr stage, median (IQR)	2 (2, 2)	2 (2, 2)	1.0
*Current DSM-IV diagnosis*^c^, *n* *(%)*			
Panic disorder with agoraphobia	0 (0%)	1 (3.2%)	1.0
Agoraphobia without history of panic disorder	8 (26.7%)	13 (41.9%)	0.28
Social phobia	2 (6.7%)	4 (12.9%)	0.67
Generalized anxiety disorder	3 (10.0%)	3 (9.7%)	1.0
Major depressive episode	1 (3.3%)	2 (6.5%)	1.0
MDS-UPDRS item 1.4 (anxious mood)	1.4 (0.9)	1.1 (0.7)	0.23
Antidepressant use, *n* (%)	9 (30.0%)	18 (58.1%)	0.040
Benzodiazepine use, *n* (%)	2 (6.7%)	4 (12.9%)	0.67
Antipsychotic use, *n* (%)	1 (3.3%)	0 (0%)	0.49
*Outcome measures (scale range)*			
Parkinson Anxiety Scale total (0–48)	18.1 (4.3)	18.5 (5.6)	0.71
Parkinson Anxiety Scale Subscale A^d^ (0–20)	10.2 (2.3)	10.5 (2.3)	0.71
Parkinson Anxiety Scale Subscale B^d^ (0–16)	4.2 (2.3)	4.1 (2.7)	0.91
Parkinson Anxiety Scale Subscale C^d^ (0–12)	3.7 (1.8)	4.0 (2.5)	0.55
Beck Depression Inventory-II (0–63)	14.1 (5.7)	14.1 (6.2)	1.0
Montreal Cognitive Assessment (0–30)	27.4 (2.1)	27.3 (1.9)	0.88
MDS-UPDRS Part I (0–52)	11.0 (5.0)	11.1 (4.8)	0.90
MDS-UPDRS Part II (0–52)	10.0 (6.5)	11.3 (7.0)	0.48
MDS-UPDRS Part III (0–132)	23.1 (10.8)	23.5 (10.3)	0.88
MDS-UPDRS Part IV (0–24)	4.9 (3.7)	3.7 (4.1)	0.26
PDQ-39 Summary Index (0–100)	25.3 (11.1)	25.8 (12.1)	0.85
Rome III Constipation Severity Scale^e^ (0–24)	4.2 (3.6)	5.5 (4.4)	0.21

Data are *n* (%) or mean (standard deviation), unless otherwise indicated.

*PASIPD* Physical Activity Scale for Individuals with Physical Disabilities, *PD* Parkinson’s disease, *LEDD* levodopa equivalent daily dose, *IQR* interquartile range, *DSM-IV* Diagnostic and Statistical Manual of Mental Disorders, Fourth Edition, *MDS-UPDRS* Movement Disorder Society-Unified Parkinson’s Disease Rating Scale, *PDQ-39* Parkinson’s Disease Questionnaire-39.

^a^Fisher’s exact tests for categorical variables and two-sample *t*-tests for continuous variables, except for Hoehn and Yahr stage (Mann–Whitney *U* test).

^b^Based on commonly accepted conversion factors^97^.

^c^Based on the Mini International Neuropsychiatric Interview, version 6.0.0.

^d^Subscale A = persistent anxiety; subscale B = episodic anxiety; subscale C = avoidance behavior.

^e^Based on the six Rome III functional constipation questions (higher score indicates greater severity).

### Compliance

Among those who completed the trial, overall compliance with the twice-daily consumption of the probiotic or placebo (calculated as the percentage of expected doses taken based on returned sachets) was very high: mean 98.5%, SD 3.2%. No significant difference was observed between the two groups: 98.5% (SD 3.6%) in the probiotic group and 98.5% (SD 2.8%) in the placebo group (*p* = 0.96).

### Blinding assessments

James’ blinding index was 0.55 (95% confidence interval (CI): 0.41–0.69), indicating overall blinding success. Bang’s blinding index was 0.037 (95% CI: −0.27 to 0.34) for the probiotic intervention and −0.13 (95% CI: −0.45 to 0.20) for placebo, confirming successful blinding in both groups.

### Clinical outcomes—Intention-to-treat analysis

After 12 weeks, both groups showed significant improvements in PAS total scores from baseline. The adjusted mean change was −6.8 points (95% CI: −10.0 to −3.5) in the probiotic group and −8.2 points (95% CI: −11.6 to −4.9) in the placebo group (*p* < 0.001 for both). There was no significant between-group difference (adjusted mean difference 1.0 point, 95% CI: −2.9 to 4.9; *p* = 0.60) (Table 2). Significant within-group improvements were also observed for all three PAS subscales, but no significant differences were observed between groups.

**Table 2:** Primary and secondary outcomes by intention-to-treat analysis

	Probiotic group (*n* = 30)			Placebo group (*n* = 31)			Adjusted mean difference (95% CI)	*p*
	Baseline	12 weeks	Adjusted mean change (95% CI)	Baseline	12 weeks	Adjusted mean change (95% CI)		
*Primary outcome*								
Parkinson Anxiety Scale	18.1 (0.8)	11.3 (1.6)	−6.8 (−10.0, −3.5)^a^	18.5 (1.0)	10.3 (1.7)	−8.2 (−11.6, −4.9)^a^	1.0 (−2.9, 4.9)	0.60
*Secondary outcomes*								
PAS Subscale A	10.2 (0.4)	7.1 (0.9)	−3.1 (−5.0, −1.3)^b^	10.5 (0.4)	6.8 (0.9)	−3.6 (−5.4, −1.8)^a^	0.3 (−1.8, 2.3)	0.79
PAS Subscale B	4.2 (0.4)	1.9 (0.6)	−2.3 (−3.4, −1.1)^a^	4.1 (0.5)	2.2 (0.5)	−1.9 (−3.0, −0.8)^a^	−0.3 (−1.6, 1.0)	0.65
PAS Subscale C	3.7 (0.3)	2.4 (0.5)	−1.3 (−2.4, −0.2)^b^	4.0 (0.4)	1.4 (0.5)	−2.6 (−3.6, −1.6)^a^	0.9 (−0.3, 2.1)	0.12
BDI-II	14.1 (1.0)	8.4 (1.8)	−5.6 (−9.2, −2.1)^b^	14.1 (1.1)	8.4 (1.7)	−5.6 (−9.1, −2.2)^b^	0.002 (−4.0, 4.0)	1.0
IDS-C	11.6 (1.0)	10.4 (1.4)	−1.2 (−4.0, 1.7)	11.6 (1.0)	9.3 (1.4)	−2.3 (−5.1, 0.5)	1.1 (−2.3, 4.4)	0.51
QIDS-SR	6.7 (0.6)	5.9 (0.8)	−0.8 (−2.5, 0.9)	6.5 (0.5)	6.4 (0.8)	−0.007 (−1.6, 1.6)	−0.6 (−2.5, 1.4)	0.55
Fatigue Severity Scale	3.9 (0.2)	3.6 (0.3)	−0.3 (−1.0, 0.4)	4.0 (0.2)	3.2 (0.3)	−0.8 (−1.4, −0.1)^c^	0.4 (−0.4, 1.1)	0.35
Starkstein Apathy Scale	13.7 (1.0)	12.5 (1.7)	−1.2 (−4.6, 2.1)	13.7 (1.1)	13.8 (1.6)	0.01 (−3.2, 3.2)	−1.3 (−5.1, 2.6)	0.51
Montreal Cognitive Assessment	27.4 (0.4)	28.7 (0.4)	1.3 (0.4, 2.2)^b^	27.3 (0.3)	27.6 (0.4)	0.3 (−0.5, 1.2)	1.1 (0.04, 2.1)	0.043
MDS-UPDRS Part I	11.0 (0.9)	8.2 (1.0)	−2.8 (−4.9, −0.7)^b^	11.1 (0.9)	7.7 (1.0)	−3.5 (−5.6, −1.4)^b^	0.5 (−2.1, 3.1)	0.67
MDS-UPDRS Part II	10.0 (1.2)	8.2 (1.3)	−1.8 (−4.5, 0.9)	11.3 (1.3)	8.0 (1.2)	−3.3 (−5.7, −0.8)^c^	0.3 (−2.6, 3.1)	0.86
MDS-UPDRS Part III	23.1 (2.0)	24.7 (2.3)	1.6 (−3.1, 6.2)	23.5 (1.9)	27.4 (2.2)	3.8 (−0.6, 8.2)	−2.6 (−7.8, 2.5)	0.31
MDS-UPDRS Part IV	4.9 (0.7)	4.7 (0.8)	−0.2 (−1.9, 1.5)	3.7 (0.7)	3.7 (0.8)	−0.1 (−1.7, 1.5)	1.0 (−1.0, 3.0)	0.31
PDQ-39 Summary Index	25.3 (2.0)	22.9 (2.9)	−2.4 (−8.1, 3.3)	25.8 (2.2)	21.7 (2.7)	−4.2 (−9.7, 1.3)	1.2 (−5.3, 7.8)	0.71
Rome III Constipation Severity Scale	4.2 (0.7)	4.2 (0.9)	−0.02 (−1.8, 1.7)	5.5 (0.8)	3.7 (0.9)	−1.8 (−3.6, −0.007)	0.5 (−1.9, 2.8)	0.68

Data are mean (SE) or mean (95% CI). *P*-values for secondary outcomes are not adjusted for multiple comparisons and should be interpreted with caution.

*PAS* Parkinson Anxiety Scale, *BDI-II* Beck Depression Inventory-II, *IDS-C* Inventory of Depressive Symptomatology, Clinician-Rated, *QIDS-SR* Quick Inventory of Depressive Symptomatology, Self-Report, *MDS-UPDRS* Movement Disorder Society-Unified Parkinson’s Disease Rating Scale, *PDQ-39* Parkinson’s Disease Questionnaire-39.

^a^The within-group change from baseline was significant at *p* < 0.001.

^b^The within-group change from baseline was significant at *p* < 0.01.

^c^The within-group change from baseline was significant at *p* < 0.05.

Montreal Cognitive Assessment (MoCA) scores improved significantly in the probiotic group (1.3 points, 95% CI: 0.4–2.2; *p* = 0.003) but not in the placebo group (0.3 point, 95% CI: −0.5 to 1.2; *p* = 0.51), resulting in a significant between-group adjusted mean difference of 1.1 points (95% CI: 0.04–2.1; *p* = 0.043). The effect on MoCA remained significant after additionally adjusting for years of education. No other secondary outcomes showed significant between-group differences.

Two participants in the placebo group adjusted their PD medications (levodopa dosing: +150 mg at week 7 and −450 mg at week 11), and these changes did not result in a significant difference in levodopa dosing between groups (*p* = 0.057). Post-intervention physical activity levels, as assessed by the Physical Activity Scale for Individuals with Physical Disabilities, remained similar between the two groups (mean MET hour/day (SD): 19.1 (11.0) in the probiotic group and 16.3 (12.2) in the placebo group; *p* = 0.38).

### Clinical outcomes—per-protocol analysis

**Fig. 2:**
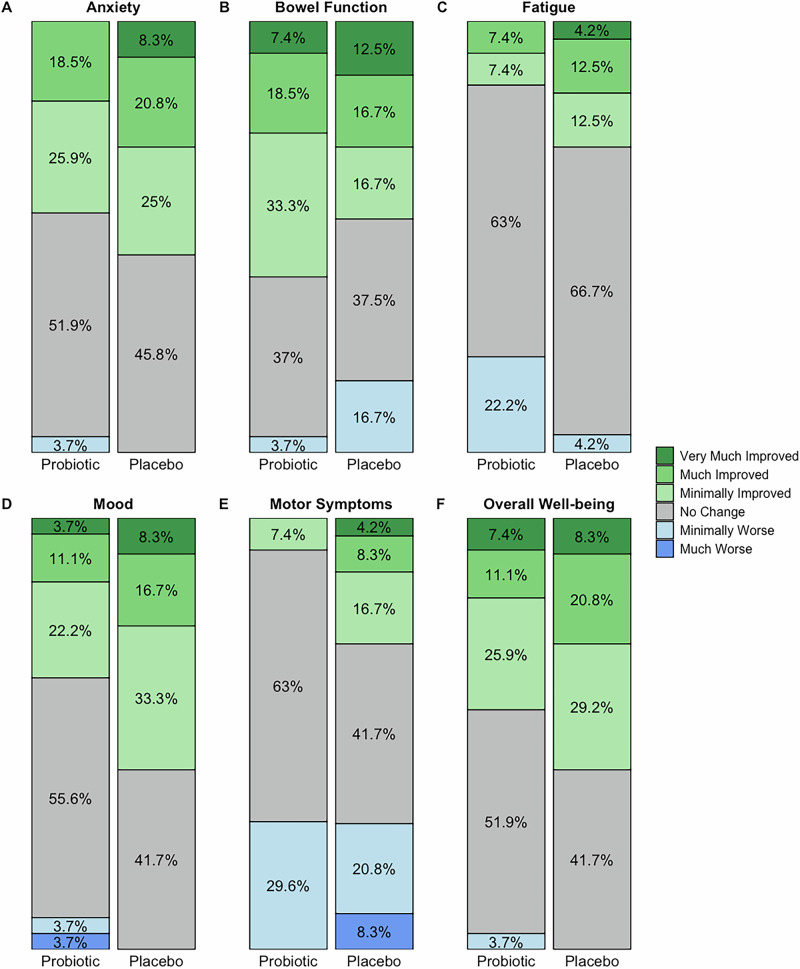
Patient Global Impression of Change ratings of trial completers. Stacked bar charts showing self-rated change across six domains for probiotic (*n* = 27) and placebo (*n* = 24) completers.

Results from per-protocol analyses were consistent with the primary ITT analysis (Supplementary Table 8). Both groups exhibited significant improvements on PAS total and subscale scores, while the probiotic group showed a significant improvement in MoCA total score not seen in the placebo group (adjusted mean difference of 1.1 points; 95% CI: 0.2–2.0; *p* = 0.016). Ratings on six domains on the Patient Global Impression of Change among completers supported these findings (Fig. 2; Table 3). Exploratory analyses of the seven MoCA domains and the Parkinson’s Disease Questionnaire-39 cognitive domain suggested a trend towards improvement in the probiotic group (Table 4).

**Table 3:** Patient Global Impression of Change scores of trial completers

Domain	Probiotic (*n* = 27)	Placebo (*n* = 24)	*p*
Anxiety	3.4 (0.8)	3.1 (1.0)	0.22
Bowel function	3.1 (1.0)	3.3 (1.3)	0.58
Fatigue	4.0 (0.8)	3.5 (0.9)	0.06
Mood	3.6 (1.0)	3.1 (1.0)	0.10
Motor symptoms	4.2 (0.6)	3.9 (1.2)	0.25
Overall well-being	3.3 (1.0)	3 (1.0)	0.30

Data are mean (SD). Lower scores indicate greater self-reported improvements. Comparisons were made using two-sample t-tests.

**Table 4:** Scores from trial completers for the seven MoCA domains and PDQ-39 cognition domain

	Probiotic (*n* = 27)		Placebo (*n* = 24)	
	Baseline	12 weeks	Baseline	12 weeks
Visuospatial/executive	4.44 (0.85)	4.67 (0.62)	4.21 (0.88)	4.42 (0.72)
Naming	2.96 (0.19)	3 (0)	2.96 (0.20)	3 (0)
Attention	5.78 (0.51)	5.89 (0.42)	6 (0)	5.71 (0.55)
Language	2.26 (0.76)	2.44 (0.75)	2.58 (0.65)	2.50 (0.59)
Abstraction	1.78 (0.51)	1.85 (0.46)	1.79 (0.51)	1.58 (0.72)
Delayed recall	4.37 (0.84)	4.52 (0.85)	3.71 (1.20)	4.29 (1.23)
Orientation	5.89 (0.32)	6 (0)	5.92 (0.28)	5.88 (0.34)
PDQ-39 cognition	25.69 (15.05)	20.14 (12.66)	25.00 (15.54)	21.35 (16.68)

Data are mean (SD). Formal statistical testing was not performed.

*MoCA* Montreal Cognitive Assessment, *PDQ-39* Parkinson’s Disease Questionnaire-39.

### Microbiome data

**Fig. 3:**
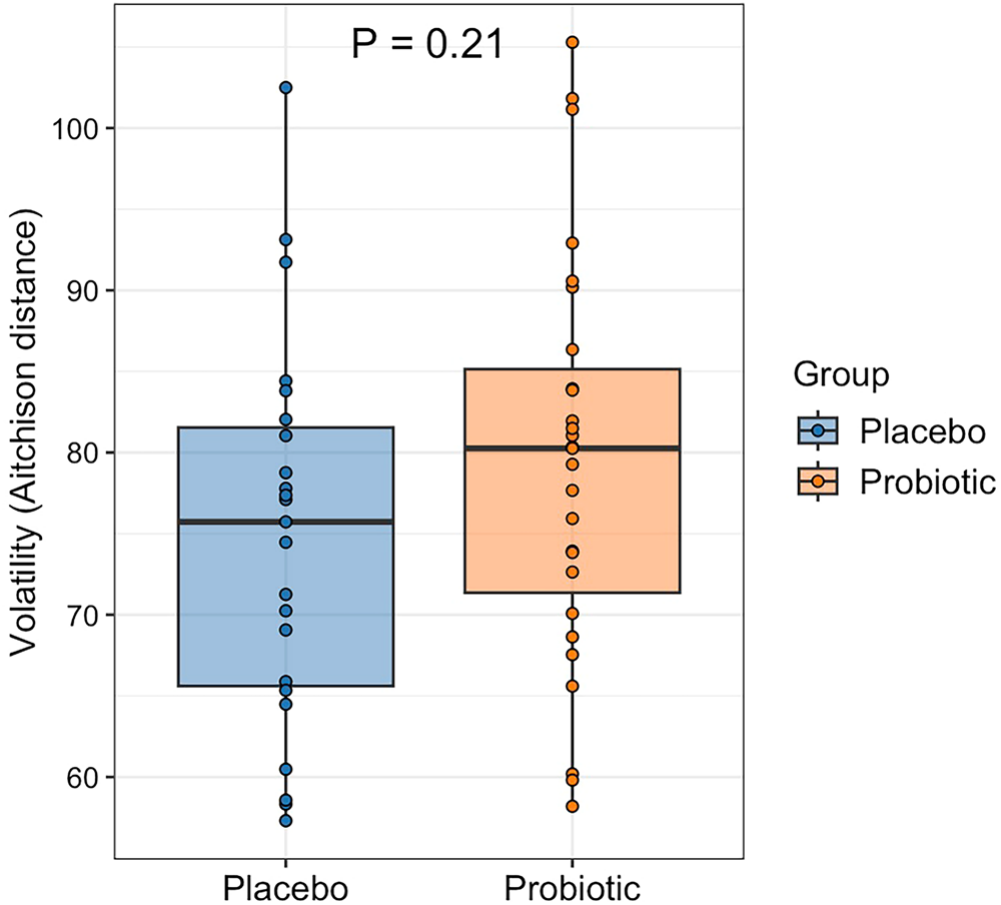
Microbiome volatility over the 12-week intervention period. Box plots showing Aitchison distance between pre- and post-intervention stool samples as a measure of compositional change in the probiotic and placebo groups.

A total of 50 participants (27 probiotic, 23 placebo) provided both pre- and post-intervention stool samples for shallow shotgun sequencing (Supplementary Fig. 1). No significant differences were observed in alpha diversity (Wilcoxon rank-sum test, all *p* > 0.05) or beta diversity (permutational multivariate analysis of variance (PERMANOVA), *p* > 0.05) between pre- and post-intervention samples within each group or between the probiotic and placebo groups. Volatility analysis revealed a marginally higher degree of compositional change in the probiotic group, although this difference was not statistically significant (Fig. 3).

The probiotic mixture contained nine strains from seven species, but differential abundance analysis was conducted at the species level due to limited strain-level sequencing resolution. Differential abundance testing showed no significant differences between groups at either the pre- or post-intervention time points (all *q* > 0.1). Similarly, pairwise analyses within each group revealed no significant changes in taxon abundance over time.

**Fig. 4:**
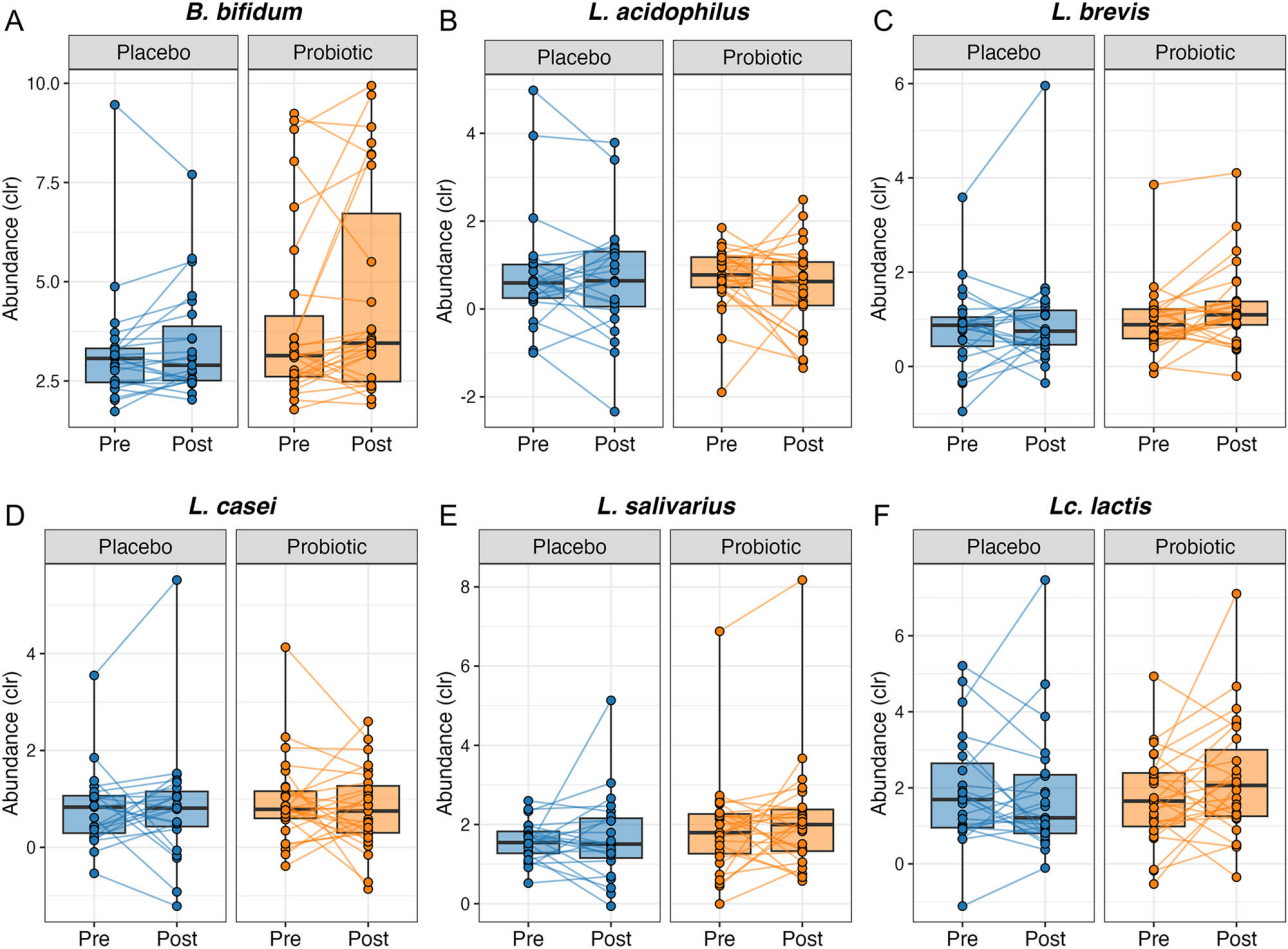
Abundances of the administered strains. Centered log-ratio (clr)-transformed abundances of six detected probiotic species: Bifidobacterium bifidum (**A**), *Lactobacillus acidophilus* (**B**), *Levilactobacillus brevis* (**C**), *Lacticaseibacillus casei* (**D**), *Ligilactobacillus salivarius* (**E**), and *Lactococcus lactis* (**F**) showed no significant changes following the 12-week probiotic intervention. Box plots depict the medians and interquartile ranges; lines connectindividual pre- and post-intervention values.

All probiotic species except *Bifidobacterium lactis* were detected. The centered log-ratio (clr)-transformed abundances of *Lc. lactis*, *L. brevis*, and *B. bifidum* showed slight, although non-statistically significant, increases following probiotic treatment (Fig. 4A–F). Of note, despite the lack of statistical significance after FDR correction, the two species with the greatest changes in abundance after 12 weeks in the probiotic group were *B. animalis* and *L. paracasei* (both increased), whereas in the placebo group, they were *Anaerococcus obesiensis* and *Haemophilus parainfluenzae* (both increased) (Supplementary Fig. 2). In addition, at the genus level, no significant differences were observed in *Lactobacillus* or *Bifidobacterium* between groups or across time points (all *q* > 0.1).

### Serum inflammatory markers

IL-1β and IL-4 were excluded from analyses, as most readings fell below detection limits (96% and 82%, respectively). Two participants did not provide post-intervention blood samples and were excluded from analysis. Additionally, two participants in the probiotic group, whose cytokine levels were several-fold above the group median at both time points, were excluded from further analysis, leaving an analyzed sample of 47 participants (24 probiotic and 23 placebo). Overall, no significant between-group differences were detected in the eight cytokines measured (Table 5).

**Table 5:** Serum inflammatory markers before and after the intervention

	Probiotic group (*n* = 24)			Placebo group (*n* = 23)			Adjusted mean difference (95% CI)	*p*
	Baseline	12 weeks	Adjusted mean change (95% CI)	Baseline	12 weeks	Adjusted mean change (95% CI)		
IL-12p70 (pg/mL)	0.5 (0.2)	0.3 (0.1)	−0.2 (−0.3, 0.01)	0.2 (0.04)	0.4 (0.1)	0.3 (0.1, 0.4)	−0.1 (−0.3, 0.1)	0.25
IL-5 (pg/mL)	0.3 (0.05)	0.3 (0.1)	0.01 (−0.1, 0.1)	0.1 (0.02)	0.3 (0.05)	0.2 (0.1, 0.2)	−0.01 (−0.1, 0.1)	0.83
IFNγ (pg/mL)	0.1 (0.03)	0.4 (0.5)	0.3 (−0.8, 1.3)	0.1 (0.01)	0.1 (0.5)	0.1 (−0.9, 1.1)	0.2 (−1.0, 1.5)	0.70
IL-6 (pg/mL)	2.4 (0.6)	1.9 (0.6)	−0.4 (−1.6, 0.8)	0.9 (0.1)	2.2 (0.6)	1.4 (0.3, 2.5)	−0.3 (−1.7, 1.1)	0.67
IL-8 (pg/mL)	12.2 (0.8)	13.3 (2.8)	1.2 (−4.4, 6.7)	13.5 (1.9)	16.3 (2.5)	2.8 (−2.2, 7.8)	−2.9 (−9.1, 3.2)	0.34
IL-22 (pg/mL)	1.1 (0.2)	1.2 (0.2)	0.1 (−0.2, 0.5)	1.0 (0.2)	1.0 (0.2)	0.02 (−0.3, 0.4)	0.2 (−0.2, 0.6)	0.36
TNFα (pg/mL)	0.8 (0.1)	0.8 (0.1)	0.04 (−0.1, 0.2)	0.6 (0.04)	0.8 (0.1)	0.2 (0.1, 0.3)	0.02 (−0.1, 0.2)	0.79
IL-10 (pg/mL)	0.6 (0.1)	0.7 (0.3)	0.1 (−0.4, 0.7)	0.7 (0.2)	0.9 (0.2)	0.2 (−0.3, 0.7)	−0.1 (−0.8, 0.5)	0.66

Data are mean (SE) or mean (95% CI). Results were obtained from an analysis of covariance, adjusted for baseline value of the cytokine, levodopa equivalent daily dose, use of antidepressant and anxiolytic medications, and baseline Rome III Constipation Severity Scale score.

### Adverse events

No serious AEs were recorded. The probiotic treatment was safe compared with placebo, with no significant difference in the number of participants reporting AEs deemed possibly, probably, or definitely related to the intervention (probiotic: 10 of 30 participants (33.3%) vs. placebo: 12 of 31 participants (38.7%); *p* = 0.79). Most AEs were related to gastrointestinal symptoms in both groups. Details on AEs are listed in Supplementary Table 9.

## Discussion

To the best of our knowledge, the TAP trial represents the first RCT specifically designed to evaluate the effects of probiotic supplementation on PD-related anxiety. We tested a multi-strain probiotic selected for its safety profile and previously demonstrated psychiatric benefits in RCTs involving non-PD populations^36–38^. In this 12-week trial involving 61 PwP with clinically significant anxiety, both the probiotic and placebo groups demonstrated substantial within-group improvements on the PAS, with no evidence of a between-group difference. Similar patterns were observed on depression measures. These findings were corroborated by patient-rated global impressions of change, which indicated comparable perceived improvements across groups. Indeed, placebo response (the overall clinical improvement seen following administration of placebo) is particularly strong and a well-documented phenomenon in interventional studies for both PD and anxiety^39–41^. The within-group improvements seen in both arms may be attributed to the broader contextual and non-specific effects, such as regression to the mean, natural symptom fluctuations, and the Hawthorne effect^41,42^. In the context of anxiety specifically, regular contact with the study team and heightened symptom awareness might have further contributed to improvement in both groups.

Generally consistent with our findings, improvements in anxiety have also been reported in the control arms of existing RCTs for anxiety in PD. In the CBT trial^17^, both participants receiving 10 weekly CBT sessions and those assigned to clinical monitoring alone demonstrated clinically relevant post-treatment improvements on the PAS and Hamilton Anxiety Rating Scale (HARS). However, greater benefits of CBT were observed on the PAS, particularly for the episodic anxiety and avoidance behavior subscales, with effects sustained at 3- and 6-month follow-ups. Similarly, in the acupuncture trial, both the real and sham groups showed clinically relevant improvements on the HARS at the end of the 8-week treatment period, whereas a between-group difference emerged at the 8-week follow-up rather than at post-treatment^18^. In the 12-week buspirone trial, which was designed to assess safety and tolerability and included a small placebo arm, reductions on the PAS and HARS of similar magnitude were observed in both the active and placebo groups^16^.

Taken together, these findings suggest that contextual and non-specific improvements are common in the control arms of PD anxiety trials, and that treatment-specific effects, when present, may emerge more clearly on selected domains or with longer follow-up. While our sample size and 12-week intervention duration are comparable to prior PD anxiety trials and most probiotic RCTs in PD^35^ and anxiety or mood disorders^33,34^, this timeframe may be insufficient to detect treatment-specific effects on neuropsychiatric outcomes. In contrast, probiotic effects on gastrointestinal outcomes have been observed over shorter intervention periods (e.g., 4 weeks)^21,25^. Future studies using a three-arm design with an untreated control group, a longer intervention period, and extended follow-ups are needed to better delineate probiotic-specific effects on anxiety outcomes in PD^42^.

Among the secondary outcomes, a significant 1.1-point improvement on the MoCA was observed in the probiotic group, along with a trend towards improvement across all tested MoCA domains. These findings need to be interpreted with caution, as the MoCA was not a primary outcome and the baseline scores were high in both groups, with 26 probiotic and 25 placebo participants scoring ≥ 26. The cohort’s high educational attainment (mean 16 years of education) may have further contributed to ceiling effects and limited sensitivity to change. Learning effects also cannot be fully minimized despite the use of alternate MoCA versions. The clinical significance of this 1.1-point change on the MoCA remains uncertain in early- to mid-stage PD, as the minimal clinically important difference (MCID) has not yet been established. In other neurological conditions, such as stroke, MCID estimates for the MoCA range from 1 to 2 points depending on the methods used, while the minimal detectable change has been reported to be substantially larger (about 5 points)^43^. The latter reflects the smallest change beyond measurement error. Although these estimates cannot be directly applied to PD, they provide context for interpreting our observed group-level changes, which should be considered preliminary and hypothesis-generating.

With these factors considered, the cognitive findings nevertheless remain intriguing given the emerging evidence linking cognition with gut microbiota in neurodegenerative disorders such as Alzheimer’s disease^44^, as well as in healthy aging^45,46^. Meta-analyses of probiotic RCTs in these populations suggest potential cognitive benefits, though substantial heterogeneity exists due to variations in probiotic strains, dosages, and study designs^47–49^. In PD, evidence for probiotic effects on cognition remains preliminary, as these have only been assessed as secondary outcomes, with three RCTs reporting slight improvements on the MoCA^31^, the Mini-Mental State Examination^27^, and the Parkinson’s Disease Questionnaire-39 cognitive domain^30^ following 12 weeks of probiotic supplementation.

Notably, the same multi-strain probiotic used in our study has been investigated in individuals with clinical depression^50^ and those with bipolar or schizophrenia spectrum disorders^51^. While no differences were observed on anxiety or mood measures compared with placebo, significant improvements were noted in cognitive reactivity (the tendency to think negatively in response to a sad mood and a strong predictor of depression)^50^ as well as in verbal memory, problem-solving, and processing speed^51^ after 8 and 12 weeks of probiotic supplementation, respectively. Similar effects on cognitive reactivity to sad mood have been reported in an RCT of the same probiotic formulation involving healthy individuals^37^, alongside another RCT that noted improved stress-related working memory, which was associated with decreased lateral prefrontal cortex activation during cognitive control^38^. Recent findings also suggest that this probiotic may enhance executive function under high cognitive load and reduce cognitive biases such as hopelessness, rumination, and aggression in healthy older adults, with acute improvements in reaction time observed within 24 h of a single dose^52^. Together, these preliminary findings support future appropriately powered RCTs that incorporate a comprehensive neuropsychological test battery as well as PD-specific cognitive scales, namely, the Parkinson’s Disease-Cognitive Rating Scale^53^ and the Parkinson’s Disease-Cognitive Functional Rating Scale^54^.

No significant probiotic-associated shifts in the gut microbiome or inflammatory cytokines were observed compared with placebo. Among the dozen probiotic RCTs in PD, microbiome changes were only assessed in four studies^26–28,32^. Findings related to inflammatory biomarkers varied widely across studies, given the range of analytes and sample types (e.g., serum, plasma, fecal) assessed, with low-quality evidence suggesting a reduction in serum inflammatory markers^35^. Our results overall align with previous RCTs in PD^26–28,32^ and with the depression trial using the same probiotic^50^, where overall diversity and structure of the microbiota remained largely unchanged despite symptom improvement, with observed effects limited to specific taxa. In our study, modest increases in volatility (i.e., compositional change over time) and in the abundances of three administered species (*B. bifidum*, *L. brevis*, and *Lc. lactis*) were observed in the probiotic group.

The second fecal samples were collected while participants were still on the probiotic to capture any transient effects before strains were washed out, given that most probiotics are detectable in stool for only 3–6 days after cessation and rarely persist beyond 1–2 weeks^55,56^. The adult gut microbiota is relatively stable and resilient, shaped largely by long-term diet and lifestyle factors, and exhibits colonization resistance that limits the establishment of ingested strains^57,58^. Consequently, probiotic effects may occur via transient interactions or functional modulation rather than lasting changes in community composition^55,57^. Factors such as strain specificity, dosage, gut transit time, and baseline microbiota can all influence persistence^56,57^. Overall, our findings align with this broader literature.

Three of the seven probiotic species (*B. bifidum*, *L. casei*, and *L. acidophilus*) have, in varied combinations, been associated with cognitive benefits in people with Alzheimer’s disease^48,49^ with animal studies suggesting that these species modulate pathways implicated in cognitive dysfunction^59,60^. As mentioned above, the mechanisms of action of probiotics are complex and highly strain- and host-dependent. Their effects can occur without direct alterations in microbiota composition or immune modulation, such as through regulation of mucin expression^61^ or enhancement of gut epithelial barrier function by breaking down lipopolysaccharide, as has been shown in vitro by the probiotic used in this study^62^. Furthermore, the increases in *B. animalis* and *L. paracasei* seen in the probiotic group are intriguing (Supplementary Fig. 2), as they belong to the same genera as the strains in the formulation (*Bifidobacterium* and *Lacticaseibacillus*) and are commonly used in both commercial probiotic products and research settings. Their proliferation suggests a potential role for cross-feeding interactions or other indirect effects of the intervention, such as modulation of microbial metabolism^63^. It is possible that longer interventions may be needed to induce more pronounced microbial shifts between groups, especially considering the broad microbiota disruption seen in PD^64^.

In addition, there have been growing validity concerns regarding the use of maltodextrin, a starch-derived polysaccharide commonly used as a control or placebo in probiotic and dietary intervention studies^65,66^. A 2022 systematic review of 70 RCTs in which maltodextrin was used as a placebo or control reported maltodextrin-associated effects in 42 studies, including changes in gut microbiota composition (50%), physiological parameters (38%), and immunological or inflammatory markers (26%), with substantial variability depending on dose, duration, and study population^66^. These findings suggest that maltodextrin may not be as biologically inert as presumed and could have contributed, at least in part, to overall compositional shifts and the gastrointestinal-related AEs observed in our placebo group. Notably, similar gastrointestinal AEs were also reported in the placebo arms of other trials investigating the same probiotic formulation^50,67,68^. Given the use of maltodextrin in other PD trials as well^25,27,29–31^, these findings collectively highlight the importance of considering its potential biological activity when interpreting changes in microbiome and gastrointestinal outcomes.

To our knowledge, this is the first RCT to investigate the effects of probiotics on anxiety symptoms in PD. Using a PD-specific anxiety measure (PAS) as the primary outcome measure offers psychometric advantages over general anxiety rating scales used in other PD trials^17,69^, particularly as it has been validated for use among Canadian PwP^70^. While the dropout rate was slightly higher than expected, our sample size and treatment duration aligned with most anxiety trials in PD^16–18^ as well as probiotic trials in both PD^35^ and non-PD^33,34^ populations. Compliance was high, and blinding success was confirmed using established indices. Importantly, no increased risks of AEs related to probiotics were noted. A notable strength of this study is the incorporation of metagenomic sequencing and inflammatory cytokine profiling.

Several important limitations should be acknowledged. First, we used three criteria to define clinically significant anxiety, which may have introduced selection bias. However, 88.5% (54 of 61 participants) of the trial cohort met the PAS threshold. As anxiety in PD is heterogenous and is not well captured by standard diagnostic categories^1,3^, this design was intentionally adopted to recruit PwP experiencing clinically meaningful anxiety symptoms, thereby enhancing the clinical relevance of the cohort and generalizability of the findings. Second, our cohort consisted predominantly of males and Caucasians, reflecting the demographics of PwP typically seen at tertiary centers and enrolled in trials^71,72^. Sex differences in anxiety symptoms have been reported by some studies in PD^73,74^; future trials in more sex-balanced cohorts will be important. In addition, this study was conducted during the COVID-19 pandemic, during which a few participants reported infection, leading to one withdrawal. The pandemic and associated restrictions may have influenced outcome measures, particularly those related to anxiety, mood, and overall well-being. To address this, participants were asked during the MINI whether the pandemic had exacerbated their anxiety, and none reported a significant impact. Additionally, two participants in the placebo group had minor PD medication adjustments, although we do not believe this influenced our results, as randomization should have ensured potential changes between both groups.

Participants were also instructed to maintain their daily routines, with a willingness to maintain habitual physical activity as an eligibility criterion. Physical activity levels showed no significant differences within or between groups pre- and post-intervention. While baseline dietary intake was well matched using the 165-item, past-month version of the Canadian Diet History Questionnaire II with portion sizes^75^, post-intervention assessments were not conducted to minimize participant burden. Although longitudinal cohort studies suggest that dietary patterns in older adults remain relatively stable over time^76–78^, unmeasured changes in dietary intake during this trial could have influenced the microbiota composition. Future probiotic RCTs would benefit from repeated assessments of lifestyle factors to better account for their influence on microbiome composition and treatment response.

In conclusion, while no group differences were observed for neuropsychiatric symptoms following the 12-week intervention, the potential cognitive benefits seen in the probiotic group warrant further investigation. With growing interest in microbiota-targeted interventions for PD, future animal and human studies should refine probiotic interventions by extending treatment durations, incorporating multi-omics approaches to identify mechanistic pathways, and evaluating individual variations in microbiota responses to better understand host-microbe interactions.

## Methods

### Study design and participants

The Treating Anxiety in Parkinson’s Disease with a Multi-Strain Probiotic (TAP) trial was a 12-week, investigator-initiated, randomized, double-blind, placebo-controlled trial conducted at the University of British Columbia (UBC) Movement Disorders Clinic in Vancouver, Canada. The study was approved by the UBC Clinical Research Ethics Board (Protocol #H18-03083) and registered on ClinicalTrials.gov (NCT03968133) on May 28, 2019, prior to the start of recruitment. Authorization was obtained from Health Canada (NOA #246158). The study was conducted in accordance with the Declaration of Helsinki and Good Clinical Practice guidelines. All participants provided electronic informed consent through the Research Electronic Data Capture (REDCap) system prior to any screening procedures. On-site monitoring visits for regulatory compliance, protocol adherence, and data completeness were conducted by a contract research organization (Allphase Clinical Research).

Participants were primarily recruited from the patient database of the UBC Movement Disorders Clinic. Additional recruitment efforts were made through advertisements at three regional movement disorders clinics, general neurology clinics, PD organizations such as the Canadian Open Parkinson Network, Parkinson Canada, and Parkinson Society British Columbia, as well as through online platforms.

Eligible participants were aged 40–80 years and had a clinical diagnosis of PD confirmed by a movement disorders specialist, in accordance with the UK Brain Bank Criteria^79^. All participants were in Hoehn and Yahr stages 1–3 and had to be willing to maintain their baseline physical activity levels throughout the study period, given the known impact of exercise on psychiatric disorders and the gut microbiota^80^. The presence of clinically significant anxiety was defined by one or more of the following in the ON medication state: (1) score ≥ 14 on the self-rated version of the PAS and/or (2) score ≥ 2 on item 1.4 (anxious mood) of the Movement Disorder Society-Unified Parkinson’s Disease Rating Scale (MDS-UPDRS), and/or (3) a clinical diagnosis of an anxiety disorder based on the Mini International Neuropsychiatric Interview (MINI) version 6.0.0. Key exclusion criteria included: (1) MoCA score < 21; (2) Beck Depression Inventory-II (BDI-II) score > 28; (3) any use of probiotics (including *Saccharomyces boulardii*) or antibiotics in the past 3 months; (4) concurrent psychotherapy or brain stimulation for mood disorders; (5) a change in antidepressant or anxiolytic medication (including benzodiazepines) within the last 4 weeks; (6) a change in PD medication within the last 2 weeks; and (7) concurrent treatment for PD with Duodopa or deep brain stimulation. A complete list of eligibility criteria is provided in Supplementary Table 1. Participants were asked to remain on stable doses of all medications throughout the trial unless changes were medically necessary.

### Randomization and masking

Participants were randomly assigned (1:1) to the probiotic or placebo group. The randomization list was generated by Winclove Probiotics (the Netherlands) using permuted blocks, with a block size of 4. All participants, research staff, and investigators were blinded to treatment allocation. Unblinding occurred following the final participant’s 30-day follow-up on July 4, 2023. As part of post-intervention assessments, participants were asked to guess their treatment allocation. Blinding success was assessed using two established indices: (1) James’ blinding index^81^, which ranges from 0 (complete unblinding) to 1 (complete blinding), with 0.5 indicating random guessing. Blinding is considered unsuccessful if the upper bound of the 95% CI is <0.5. (2) Bang’s blinding index^82^, calculated per treatment arm, ranges from −1 to 1, where values between −0.2 and 0.2 indicate successful blinding. Further, blinding is considered unsuccessful if the one-sided 95% CI does not include 0. Both indices were analyzed with the R package *BI* (v1.2.0).

### Intervention

The probiotic group received sachets containing 2 g of freeze-dried probiotic powder (Ecologic^®^ Barrier, Winclove Probiotics), composed of a carrier mixture of maize starch and maltodextrin, and nine bacterial strains: *Bifidobacterium bifidum* W23, *Bifidobacterium lactis* W51, *Bifidobacterium lactis* W52, *Lactobacillus acidophilus* W37, *Levilactobacillus brevis* W63 (formerly known as *Lactobacillus brevis* W63), *Lacticaseibacillus casei* W56 (formerly known as *Lactobacillus casei* W56), *Ligilactobacillus salivarius* W24 (formerly known as *Lactobacillus salivarius* W24), *Lactococcus lactis* W19, and *Lactococcus lactis* W58.

This probiotic formulation was selected based on available scientific evidence at the time of trial design, including in vitro evidence for its ability to enhance gut barrier function as measured with transepithelial electrical resistance^62^; preclinical studies showing improvements in depressive-like behavior in rats^83,84^; and RCT data demonstrating reductions in anxiety and fatigue^36^, improved cognitive reactivity to sad mood^37^ and modulation of acute stress responses^38^, all with a good safety profile.

Sachets were taken twice daily (total daily dose: 1 × 10^10^ colony-forming units (CFU); each gram contained 2.5 × 10^9^ CFU). Placebo sachets contained only the carrier mixture and were identical in color, taste, and smell to the probiotics. Participants were instructed to dissolve the powder in room-temperature water and consume it twice daily at consistent times—at least 30 min before breakfast and before bedtime. Participants were asked to return all used and unused sachets for compliance checks. Compliance was assessed by counting unused sachets to determine missed doses, verified by the number of used sachets returned, and expressed as the proportion of expected doses taken.

### Procedures

As the study was conducted during the COVID-19 pandemic, a hybrid model incorporating remote and in-person procedures was implemented to minimize travel. Prospective participants underwent pre-screening, and those interested and potentially eligible were scheduled for a remote consenting session. Following electronic consent, demographic and clinical details were collected, and remote psychiatric assessments, including the MINI, PAS, and BDI-II, were conducted. The MINI was administered via videoconferencing by one of the study team physicians (N.J.A., D.M., and F.P.).

Participants with clinically significant anxiety but without severe comorbid depression proceeded to an in-person enrollment visit (week 0), where they completed additional baseline assessments. A list of assessments can be found in Supplementary Table 2. Final eligibility criteria checks were conducted at the visit. Participants were instructed to collect a baseline stool sample within one week prior to initiating the intervention (week 1). Remote phone check-ins for compliance and adverse events (AEs) occurred following completion of weeks 4 and 8. The post-intervention visit was held at week 13 (i.e., after 12 weeks of intervention), with the final probiotic or placebo dose taken that morning. Where feasible, the second visit was scheduled at a similar time of day as the baseline visit to minimize diurnal variation in outcome measures. A follow-up call was conducted 30 days later. All assessments were conducted in the ON medication state.

### Outcomes

The primary outcome was the between-group difference in the PAS total score after the 12-week intervention. The 12-item self-rated PAS is a validated measure of anxiety symptom severity in PwP^69^, including validation in Canadian cohorts^70^. Secondary outcomes included the three PAS subscales, BDI-II, MoCA, MDS-UPDRS, and Parkinson’s Disease Questionnaire-39 (Supplementary Table 2). Alternate versions of the MoCA (versions 7.1, 7.2, and 7.3) were used across study visits to minimize learning effects. At baseline, participants with documented prior MoCA exposure in clinical records were administered an alternate version; otherwise, version 7.1 was used. At post-intervention, a different version from that used at baseline was administered, selected at random from the remaining versions. Changes in gut microbiota and serum inflammatory markers were examined on an exploratory basis.

### Biological sample collection

Serum samples were collected at the two in-person visits and were stored at −80 °C, as previously described^85^. At enrollment, participants received fecal sample collection kits containing OMNIgeneGUT (OM-200; DNA Genotek Inc., Canada) and were instructed to collect and mail their first sample within one week. The second sample was collected after the 12-week intervention, while participants were still on the assigned product and before the final in-person visit. Fecal samples were aliquoted upon receipt and stored at −80 °C. All serum and fecal aliquots were thawed once for analysis.

### Sample size calculation

As no RCTs with anxiety as the primary outcome in PD were published at the time of designing the TAP trial, the power calculation was based on validation studies of the PAS^69,86^. Power calculation was based on a standardized difference of 0.80 in PAS total score, with alpha set at 0.05 and power set at 0.80. The difference of interest (i.e., the mean score difference between patients without anxiety and those with mild-to-moderate anxiety) was set at 6 points^69^, with an estimated standard deviation of 7.5^69,86^. This yielded a required sample size of 52 (26 per group). A sample size of 30 patients per group would account for an expected drop-out rate of 15% based on a probiotic RCT for PD-related constipation^21^.

### Cytokine measurements and analysis

Serum samples were analyzed using the CorPlex Human Cytokine 10-plex (cat ID 85-329, lot 504217) on the Simoa SP-X platform (Quanterix, USA). This panel measured IL-12p70, IL-1β, IL-4, IL-5, IL-6, IL-8, IL-10, IL-22, TNFα, and IFNγ. Assays were performed according to the manufacturer’s instructions, with laboratory personnel blinded to group allocation. Each run included an 8-point calibrator curve and three plasma samples from healthy individuals as inter-run matrix controls. Pre- and post-intervention samples from each participant were kept together, with an equal mix of groups randomly assigned across the three plates. Samples were assayed in duplicate. Quality control measures are summarized in Supplementary Table 3. The average of the values was used for analysis. Statistical analyses followed the approach used for clinical outcomes.

### Microbiome sequencing and processing

Shallow shotgun sequencing was commercially performed on 111 fecal samples (61 pre- and 50 post-intervention) by Microbiome Insights (Richmond, Canada), with personnel blinded to treatment allocation. DNA was extracted using the QIAGEN MagAttract PowerSoil DNA KF Kit (formerly MO BIO PowerSoil DNA Kit) using a KingFisher robot. DNA quality was assessed via gel electrophoresis and quantified using a Qubit 3.0 fluorometer (Thermo Fisher Scientific, USA). Libraries were prepared using an Illumina Nextera library preparation kit with an in-house protocol (Illumina, USA). Paired-end sequencing (2 × 150 bp) was done on a NextSeq 500.

Quality evaluation was performed using FastQC (v0.11.5). Raw sequences were processed in four steps: adapter sequences were removed using cutadapt (v2.6)^87^, followed by read trimming with Trimmomatic (v0.36)^88^ using the parameters LEADING:3, TRAILING:3, SLIDINGWINDOW:4:15, and MINLEN:36. Next, low-complexity reads were removed using Komplexity (v0.3.6)^89^. Lastly, host sequences were filtered out by removing reads that mapped to the Genome Reference Consortium Human Reference 37. The remaining reads were taxonomically classified using Kraken2 with the PlusPF database from 2021-05-17^90^. Following pre-processing, the average number of bacterial reads per sample was 1,726,128 (median: 1,631,310), ranging from 524,167 to 4,138,797. Taxa that were unknown at the species level were not considered in downstream analysis.

### Microbiome data analysis

All analyses were performed in R (v4.4.2). Stacked bar plots were generated by normalizing counts to relative abundances, with each sample summing to 1. Taxa detected at less than 1% relative abundance across samples were aggregated as rare taxa solely for the bar plots. As ratios are invariant to subsetting and given our use of compositional data analysis techniques^91^, taxa present in less than 5% of samples at the species level were excluded from all analyses except alpha diversity analysis. Alpha diversity was assessed using Chao1, Shannon entropy, and Simpson index, computed using *iNEXT* (v3.0.1)^92^. Differences between groups were tested with the Wilcoxon rank-sum test. Clr transformation was applied to the count table, with zeroes imputed using the *const* method^93^. Beta diversity was computed in terms of Aitchison distance, and differences were assessed using PERMANOVA implementation from *vegan* (v2.6-10) with 9999 permutations. Differential abundance of taxa was assessed using linear models on the clr-transformed data. False discovery rate (FDR) correction using the Benjamini–Hochberg procedure was performed, with a *q*-value of 0.1 used as a cut-off^91^. Microbial volatility was quantified as the Aitchison distance between pre- and post-intervention samples using *volatility* (v0.0.9)^94^.

### Statistical analysis of clinical outcomes

A prespecified analysis of covariance was used to analyze the effects of treatment on the primary and secondary outcomes. Covariates included the baseline values of the dependent variables, levodopa equivalent daily dose, use of antidepressant and anxiolytic medications, and baseline Rome III Constipation Severity Scale score. Analyses followed an ITT approach. Data were assumed to be missing at random, as participants who withdrew tended to show more severe baseline clinical characteristics, such as higher MDS-UPDRS Parts I, II, and IV scores (Supplementary Table 4). These observations suggest that missingness was related, at least in part, to observed variables, supporting the plausibility of the missing at random assumption^95^. Multiple imputation was therefore performed to handle missing post-intervention data using the R package *mice* (v3.17.0)^96^. Twenty-five imputed datasets were generated separately for each group using the predictive mean matching method with 25 iterations, incorporating demographic, clinical, and baseline values of the outcomes as predictors. Diagnostic checks ensured convergence and plausibility of imputed values. Within-group changes from baseline were estimated using the same models, with outcomes and baseline measures centered by the grand baseline mean. All statistical analyses were conducted in R using *stats* (v4.4.2) for fitting the general linear models and *emmeans* (v1.10.5) for calculating the adjusted outcome values, standard errors (SEs), and 95% CIs. An additional per-protocol analysis included participants who completed the trial. No multiplicity adjustments were applied to secondary outcomes. Significance was established a priori as a *p*-value < 0.05.

## Supplementary information

Revised TAP - Supplementary Materials January 2026

## Data Availability

The data that support the findings of this study are available on request from the corresponding author (S.A.C.). The data are not publicly available due to privacy or ethical restrictions.

## Code availability

No custom codes were used. Microbiome data analysis followed the methods detailed in ref. ^91^.
