# Supplementary material for: Probiotic supplementation for anxiety symptoms in people with Parkinson’s disease: a randomized, double-blind, placebo-controlled trial
Source: NPJ Parkinsons Dis. 2026 Apr 25;12:104. doi: 10.1038/s41531-026-01364-1 (PMC13125604; doi:10.1038/s41531-026-01364-1)
Supplement: Supplementary file 1 — Revised TAP - Supplementary Materials January 2026 [file 41531_2026_1364_MOESM1_ESM.pdf]

Probiotic supplementation for anxiety symptoms in people with Parkinson's disease: a randomized, double-blind, placebo-controlled trial

Supplementary Materials

CONSORT 2025 Checklist. 2
Supplementary Table 1. Inclusion and exclusion criteria. 5
Supplementary Table 2. Assessments administered at baseline and post-intervention. 6
Supplementary Table 3. Quality control and quality assurance measures for calibrators and experimental specimens. 7
Supplementary Table 4. Baseline characteristics of participants who completed the study versus those who withdrew. 8
Supplementary Table 5. Reasons for discontinuation and their relationship to the intervention. .. 9
Supplementary Table 6. Different anxiety criteria fulfilled by the 61 enrolled participants. 9
Supplementary Table 7. Additional demographic and clinical characteristics at baseline. 10
Supplementary Table 8. Primary and secondary outcomes by per-protocol analysis. 11
Supplementary Table 9. Adverse events. 12
Supplementary Figure 1. Stacked bar plot. 13
Supplementary Figure 2. Changes in the microbiota following the 12-week intervention. 14
References 15

Probiotic supplementation for anxiety symptoms in people with Parkinson's disease: a randomized, double-blind, placebo-controlled trial

CONSORT 2025 Checklist.

Section / Topic	No	CONSORT 2025 checklist item description	Reported on page no.
**Title and abstract**			
Title and structured abstract	1a	Identification as a randomised trial	1
	1b	Structured summary of the trial design, methods, results, and conclusions	1
**Open science**			
Trial registration	2	Name of trial registry, identifying number (with URL) and date of registration	1, 8
Protocol and statistical analysis plan	3	Where the trial protocol and statistical analysis plan can be accessed	10
Data sharing	4	Where and how the individual de-identified participant data (including data dictionary), statistical code and any other materials can be accessed	10
Funding and conflicts of interest	5a	Sources of funding and other support (e.g., supply of drugs), and role of funders in the design, conduct, analysis and reporting of the trial	13
	5b	Financial and other conflicts of interest of the manuscript authors	13
**Introduction**			
Background and rationale	6	Scientific background and rationale	1-2
Objectives	7	Specific objectives related to benefits and harms	8-9
**Methods**			
Patient and public involvement	8	Details of patient or public involvement in the design, conduct and reporting of the trial	8
Trial design	9	Description of trial design including type of trial (e.g., parallel group, crossover), allocation ratio, and framework (e.g., superiority, equivalence, non-inferiority, exploratory)	8-9
Changes to trial protocol	10	Important changes to the trial after it commenced including any outcomes or analyses that were not prespecified, with reason	N/A
Trial setting	11	Settings (e.g., community, hospital) and locations (e.g., countries, sites) where the trial was conducted	8
Eligibility criteria	12a	Eligibility criteria for participants	8-9; Supplementary materials page 5
	12b	If applicable, eligibility criteria for sites and for individuals delivering the interventions (e.g., surgeons, physiotherapists)	N/A
Intervention and comparator	13	Intervention and comparator with sufficient details to allow replication. If relevant, where additional materials describing the intervention and comparator (e.g., intervention manual) can be accessed	9

Outcomes	14	Pre-specified primary and secondary outcomes, including the specific measurement variable (e.g., systolic blood pressure), analysis metric (e.g., change from baseline, final value, time to event), method of aggregation (e.g., median, proportion), and time point for each outcome	9
Harms	15	How harms were defined and assessed (e.g., systematically, non-systematically)	9
Sample size	16a	How sample size was determined, including all assumptions supporting the sample size calculation	9
	16b	Explanation of any interim analyses and stopping guidelines	N/A
Randomisation:			
Sequence generation	17a	Who generated the random allocation sequence and the method used	9
	17b	Type of randomisation and details of any restriction (e.g., stratification, blocking and block size)	9
Allocation concealment mechanism	18	Mechanism used to implement the random allocation sequence (e.g., central computer/telephone; sequentially numbered, opaque, sealed containers), describing any steps to conceal the sequence until interventions were assigned	9
Implementation	19	Whether the personnel who enrolled and those who assigned participants to the interventions had access to the random allocation sequence	9
Blinding	20a	Who was blinded after assignment to interventions (e.g., participants, care providers, outcome assessors, data analysts)	9-10
	20b	If blinded, how blinding was achieved and description of the similarity of interventions	9
Statistical methods	21a	Statistical methods used to compare groups for primary and secondary outcomes, including harms	10
	21b	Definition of who is included in each analysis (e.g., all randomised participants), and in which group	10
	21c	How missing data were handled in the analysis	10
	21d	Methods for any additional analyses (e.g., subgroup and sensitivity analyses), distinguishing prespecified from post-hoc	9-10
**Results**			
Participant flow, including flow diagram	22a	For each group, the numbers of participants who were randomly assigned, received intended intervention, and were analysed for the primary outcome	2
	22b	For each group, losses and exclusions after randomisation, together with reasons	2; Supplementary materials page 9
Recruitment	23a	Dates defining the periods of recruitment and follow-up for outcomes of benefits and harms	2, 9
	23b	If relevant, why the trial ended or was stopped	N/A
Intervention and comparator delivery	24a	Intervention and comparator as they were actually administered (e.g., where appropriate, who delivered the intervention/comparator, how participants adhered, whether they were delivered as intended [fidelity])	2
	24b	Concomitant care received during the trial for each group	3; Supplementary materials page 10

Baseline data	25	A table showing baseline demographic and clinical characteristics for each group	3; Supplementary materials page 10
Numbers analysed, outcomes and estimation	26	For each primary and secondary outcome, by group:<br>● the number of participants included in the analysis<br>● the number of participants with available data at the outcome time point<br>● result for each group, and the estimated effect size and its precision (such as 95% confidence interval)<br>● for binary outcomes, presentation of both absolute and relative effect size	4
Harms	27	All harms or unintended events in each group	4; Supplementary materials page 12
Ancillary analyses	28	Any other analyses performed, including subgroup and sensitivity analyses, distinguishing pre-specified from post-hoc	3-4
**Discussion**			
Interpretation	29	Interpretation consistent with results, balancing benefits and harms, and considering other relevant evidence	4-8
Limitations	30	Trial limitations, addressing sources of potential bias, imprecision, generalisability, and, if relevant, multiplicity of analyses	8

*We strongly recommend reading this statement in conjunction with the CONSORT 2025 Explanation and Elaboration and/or the CONSORT 2025 Expanded Checklist for important clarifications on all the items. We also recommend reading relevant CONSORT extensions. See www.consort-spirit.org.

Citation: Hopewell S, Chan AW, Collins GS, Hróbjartsson A, Moher D, Schulz KF, et al. CONSORT 2025 Statement: updated guideline for reporting randomised trials. BMJ. 2025; 388:e081123. https://dx.doi.org/10.1136/bmj-2024-081123.

© 2025 Hopewell et al. This is an Open Access article distributed under the terms of the Creative Commons Attribution License (https://creativecommons.org/licenses/by/4.0/), which permits unrestricted use, distribution, and reproduction in any medium, provided the original work is properly cited.

Probiotic supplementation for anxiety symptoms in people with Parkinson's disease: a randomized, double-blind, placebo-controlled trial

Supplementary Table 1. Inclusion and exclusion criteria.

**Inclusion criteria**
- Diagnosis of Parkinson's disease based on the UK Brain Bank Criteria
- Between ages 40-80
- Hoehn and Yahr stage between 1-3 in the ON state
- Anxiety, defined by a score of 14 or higher on the self-rated version of the Parkinson Anxiety Scale and/or a score of 2 or higher on the Movement Disorder Society-Unified Parkinson's Disease Rating Scale item 1.4 (anxious mood), and/or a clinical diagnosis of an anxiety disorder based on the Mini International Neuropsychiatric Interview in the ON state
- Women of childbearing potential agreeable to use a medically approved method of birth control (e.g. hormonal contraceptives, intrauterine devices, vasectomy/tubal ligation, barrier methods and double barrier methods) and must have negative urine pregnancy test result at screening and baseline
- Willingness to maintain current physical activity levels during study period
**Exclusion criteria**
- Atypical parkinsonism
- Active suicidality
- Active psychosis
- Montreal Cognitive Assessment score below 21 in the ON state
- Beck Depression Inventory-II score above 28 in ON state
- Probiotic supplements, *Saccharomyces boulardii*, and/or antibiotic usage in the past 3 months
- Anti-inflammatory drug usage more than twice a week (e.g. corticosteroids, naproxen, ibuprofen, celecoxib). Use of daily 81 mg acetylsalicylic acid permitted
- Use of natural health products that affect depression (e.g. St. John's wort, passion flower)
- Concurrent psychotherapy or brain stimulation for the treatment of mood disorders
- Change in antidepressant or anxiolytic medication (including benzodiazepines) within the last 4 weeks
- Change in Parkinson's medication within the last 2 weeks
- Neurological disease other than Parkinson's disease, including Alzheimer's disease, multi-infarct dementia, Huntington's disease, normal pressure hydrocephalus, a brain tumor, progressive supranuclear palsy, seizure disorder, subdural hematoma, multiple sclerosis, or history of significant head trauma
- An immune-compromised condition (e.g. AIDS, lymphoma, patients undergoing long-term corticosteroid treatment)
- A known bleeding disorder
- Current illness (e.g. a cold or flu-like symptoms) and infections (e.g. hepatitis, HIV, gastroenteritis, fungal, or parasitic infections)
- Allergy to corn starch or corn
- Concurrent treatment for Parkinson's disease with Duodopa or Deep Brain Stimulation
- Women who are pregnant, breastfeeding, or planning to become pregnant during the course of the trial
- Unstable medical conditions or serious disease/conditions (e.g. cancer, cardiovascular, renal, lung, diabetes)
- Drug and/or substance abuse

Supplementary Table 2. Assessments administered at baseline and post-intervention.

	Baseline (Week 0)	Post-intervention (Week 13)
**Participant information**		
Demographic information, medical history, family history, social history	x	
Height, weight, waist circumference	x	x
Medication review	x	x
Pregnancy test (women of childbearing potential)	x	
Canadian Diet History Questionnaire II, past month version with portion sizes[1]	x	
Caffeine Content and Intake Chart	x	
The Physical Activity Scale for Individuals with Physical Disabilities (PASIPD)[2]	x	x
**Clinical assessments and questionnaires**		
Parkinson Anxiety Scale (PAS)[3]	x	x
Mini International Neuropsychiatric Interview (MINI)[4]	x	
Beck Depression Inventory-II (BDI-II)[5]	x	x
Inventory of Depressive Symptomatology, Clinician-Rated (IDS-C)[6]	x	x
Quick Inventory of Depressive Symptomatology, Self-Report (QIDS-SR)[6]	x	x
Fatigue Severity Scale (FSS)[7]	x	x
Starkstein Apathy Scale (SAS)[8]	x	x
Montreal Cognitive Assessment (MoCA)[9]	x	x
Movement Disorder Society-Unified Parkinson's Disease Rating Scale (MDS-UPDRS)[10]	x	x
Parkinson's Disease Questionnaire-39 (PDQ-39)[11]	x	x
Rome III Bowel Disorders Module[12] (for Rome III Constipation Severity Scale[13])	x	x
Patient Global Impression of Change (PGIC)		x
Compliance Survey		x
**Biospecimen**		
Serum sample	x	x
Fecal sample	x	x

Supplementary Table 3. Quality control and quality assurance measures for calibrators and experimental specimens.

**Analyte**	**Calibrator**				**Experimental Serum**
	Inter-well CV (%)	Inter-run CV (%)	Relative error (%)	Recovery (%)	Inter-well CV (%)
hIL-12p70	13	17	4.9	101	31
hIL-5	17	6.5	5.9	101	17
hIFNγ	11	6.3	8	101	10
hIL-6	9.2	3.4	4.2	101	9.6
hIL-8	4.8	7.6	8	101	8.1
hIL-22	16	11	5	99	9.5
hTNFα	3.7	4.8	6.7	98	8.5
hIL-10	9.4	3.4	5.5	98	6.8

Abbreviation: CV, coefficient of variation.
Inter-well CV: Each calibrator/specimen is analyzed in duplicate; this represents the CV of the concentration replicates from independent wells on the same run. *This represents the variability within the specimen.*
Inter-run CV: The CV of mean concentration across independent plates/runs for calibrators/specimens included in multiple analyses. *This demonstrates the run-to-run variability.*
Relative error: The percentage difference between the calculated and theoretical concentration, based on the certificate of analysis for calibrators.
Recovery: (Calculated / Theoretical concentration) × 100%.

Supplementary Table 4. Baseline characteristics of participants who completed the study versus those who withdrew.

	Completed (n = 51)	Withdrew (n = 10)
Age, years	64.5 (7.1)	62.7 (12.1)
Sex, n (%)		
Male	36 (70.6%)	6 (60.0%)
Female	15 (29.4%)	4 (40.0%)
Education, years	16.7 (3.1)	15.9 (2.6)
Body mass index, kg/m^2	26.4 (4.5)	23.3 (2.3)
PD-related variables		
Disease duration, years	7.1 (4.6)	9.6 (6.3)
LEDD, mg	836.9 (449.1)	1258.7 (446.7)
Levodopa use, n (%)	47 (92.2%)	10 (100.0%)
Hoehn and Yahr stage, median (IQR)	2 (2, 2)	2 (2, 2)
Parkinson Anxiety Scale total	17.8 (4.6)	20.8 (5.9)
Beck Depression Inventory-II	13.5 (5.9)	17.1 (5.3)
Fatigue Severity Scale	3.8 (1.3)	4.4 (1.2)
Starkstein Apathy Scale	13.3 (5.4)	15.8 (7.1)
Montreal Cognitive Assessment	27.4 (1.7)	26.9 (3.0)
MDS-UPDRS Part I	10.5 (4.8)	13.9 (4.3)
MDS-UPDRS Part II	9.8 (6.4)	15.2 (6.8)
MDS-UPDRS Part III	23.4 (10.4)	23.2 (11.3)
MDS-UPDRS Part IV	3.6 (3.7)	7.9 (3.0)
PDQ-39 Summary Index	24.8 (11.2)	29.4 (13.1)
Rome III Constipation Severity Scale	4.7 (4.1)	5.8 (3.9)

Data are n (%) or mean (standard deviation), unless otherwise indicated.

Abbreviations: PD, Parkinson's disease; LEDD, levodopa equivalent daily dose; IQR, interquartile range; MDS-UPDRS, Movement Disorder Society-Unified Parkinson's Disease Rating Scale; PDQ-39, Parkinson's Disease Questionnaire-39.

Supplementary Table 5. Reasons for discontinuation and their relationship to the intervention.

Adverse events	Relationship to the intervention
**Probiotic group (n = 3)**	
Fall (1)	Unlikely related
Hematuria (1)	Possibly related
Nausea (1)	Probably related
**Placebo group (n = 7)**	
Abdominal cramping and diarrhea (1)	Possibly related for both
Bloating and flatulence (1)	Possibly related for both
Flatulence and dysphagia (1)	Probably related for both
COVID-19 infection (1)	Not related
Sciatica (1)	Not related
Worsening of PD symptoms and anxiety (2)	Possibly related for both

Supplementary Table 6. Different anxiety criteria fulfilled by the 61 enrolled participants.

	**PAS**	**MINI**	**MDS-UPDRS item 1.4**	**n (%)**
**Meeting one criterion**	■			28 (45.9)
		■		4 (6.6)
			■	2 (3.3)
**Meeting two criteria**	■	■		12 (19.7)
	■		■	6 (9.8)
		■	■	1 (1.6)
**Meeting all three criteria**	■	■	■	8 (13.1)
**n (%) fulfilling a criterion**	54 (88.5)	25 (41.0)	17 (27.9)	

Note: grey tile = fulfilled a particular anxiety criterion.

Abbreviations: PAS, Parkinson Anxiety Scale; MINI, Mini International Neuropsychiatric Interview; MDS-UPDRS, Movement Disorder Society-Unified Parkinson's Disease Rating Scale.

Supplementary Table 7. Additional demographic and clinical characteristics at baseline.

	Probiotic (n = 30)	Placebo (n = 31)	*p*[1]
PD-related variables			
PD age at onset, years	55.7 (7.7)	57.7 (9.8)	0.36
Entacapone use, n (%)	5 (16.7%)	4 (12.9%)	0.73
Dopamine agonist use, n (%)	5 (16.7%)	1 (3.2%)	0.10
MAO-B inhibitor use, n (%)	7 (23.3%)	3 (9.7%)	0.18
Amantadine use, n (%)	3 (10.0%)	6 (19.4%)	0.47
Family history of PD, n (%)	8 (26.7%)	8 (25.8%)	1.0
Additional outcome measures (scale range)			
IDS-C (0-84)	11.6 (5.5)	11.6 (5.3)	0.97
QIDS-SR (0-27)	6.7 (3.4)	6.5 (3.0)	0.79
Fatigue Severity Scale (1-7)	3.9 (1.3)	4.0 (1.3)	0.85
Starkstein Apathy Scale (0-42)	13.7 (5.5)	13.7 (6.0)	1.0
Dietary intake			
Total energy, kCal	2282.5 (945.4)	2020.9 (561.8)	0.19
Carbohydrates, g	267.1 (122.2)	237.5 (73.5)	0.26
Protein, g	88.4 (33.9)	82.8 (27.6)	0.48
Fat, g	94.2 (42.8)	83.0 (25.3)	0.22
Dietary fibre, g	26.3 (10.1)	24.2 (6.9)	0.33
Caffeine, mg	143.6 (103.3)	179.5 (148.8)	0.29
Irritable bowel syndrome, n (%)	3 (10.0%)	6 (19.4%)	0.47
Laxative use, n (%)	7 (23.3%)	14 (45.2%)	0.11

Data are n (%) or mean (standard deviation).

Abbreviations: PD, Parkinson's disease; MAO-B, monoamine oxidase B; IDS-C, Inventory of Depressive Symptomatology, Clinician-Rated; QIDS-SR, Quick Inventory of Depressive Symptomatology, Self-Report.

[1] Fisher's exact tests for categorical variables and two-sample t-tests for continuous variables.

Supplementary Table 8. Primary and secondary outcomes by per-protocol analysis.

	Probiotic group (n = 27)			Placebo group (n = 24)			Adjusted mean difference (95% CI)	*p*
	Baseline	12 weeks	Adjusted mean change (95% CI)	Baseline	12 weeks	Adjusted mean change (95% CI)		
**Primary outcome**								
Parkinson Anxiety Scale	18.1 (0.9)	11.2 (1.5)	-6.9 (-10.0, -3.8)[1]	17.5 (1.0)	10.3 (1.4)	-7.1 (-10.0, -4.3)[1]	0.9 (-2.5, 4.3)	0.60
**Secondary outcomes**								
PAS Subscale A	10.4 (0.4)	7.1 (0.8)	-3.2 (-5.0, -1.5)[1]	10.2 (0.5)	6.9 (0.8)	-3.3 (-4.9, -1.7)[1]	0.3 (-1.6, 2.1)	0.77
PAS Subscale B	4.3 (0.4)	1.6 (0.5)	-2.7 (-3.7, -1.6)[1]	3.5 (0.5)	2.1 (0.5)	-1.4 (-2.4, -0.5)[1]	-0.5 (-1.7, 0.7)	0.39
PAS Subscale C	3.5 (0.3)	2.4 (0.5)	-1.1 (-2.1, -0.1)[3]	3.8 (0.4)	1.4 (0.5)	-2.4 (-3.3, -1.4)[1]	1.0 (-0.1, 2.1)	0.079
BDI-II	14.1 (1.1)	8.1 (1.7)	-6.0 (-9.4, -2.7)[2]	12.8 (1.2)	8.2 (1.5)	-4.6 (-7.7, -1.5)[2]	-0.2 (-3.9, 3.6)	0.93
IDS-C	11.6 (1.1)	10.4 (1.3)	-1.2 (-3.9, 1.5)	11.8 (1.2)	9.2 (1.2)	-2.6 (-5.1, -0.1)[3]	1.2 (-1.7, 4.1)	0.42
QIDS-SR	6.3 (0.6)	5.7 (0.7)	-0.6 (-2.1, 0.8)	6.2 (0.6)	6.3 (0.7)	0.1 (-1.3, 1.4)	-0.6 (-2.2, 1.1)	0.49
Fatigue Severity Scale	3.8 (0.2)	3.6 (0.3)	-0.3 (-0.8, 0.3)	3.9 (0.3)	3.2 (0.2)	-0.7 (-1.2, -0.2)[3]	0.3 (-0.3, 0.9)	0.27
Starkstein Apathy Scale	13.7 (1.1)	12.3 (1.4)	-1.4 (-4.1, 1.4)	12.9 (1.1)	13.8 (1.3)	0.9 (-1.6, 3.4)	-1.5 (-4.5, 1.5)	0.33
Montreal Cognitive Assessment	27.6 (0.3)	28.7 (0.4)	1.1 (0.3, 1.9)[2]	27.2 (0.4)	27.6 (0.4)	0.4 (-0.4, 1.1)	1.1 (0.2, 2.0)	0.016
MDS-UPDRS Part I	10.7 (0.9)	7.5 (0.9)	-3.2 (-5.0, -1.5)[2]	10.2 (1.0)	7.9 (0.8)	-2.3 (-3.9, -0.7)[2]	-0.4 (-2.3, 1.5)	0.64
MDS-UPDRS Part II	10.0 (1.3)	7.3 (1.0)	-2.7 (-4.7, -0.6)[3]	9.5 (1.3)	8.1 (0.9)	-1.4 (-3.3, 0.5)	-0.9 (-3.1, 1.4)	0.45
MDS-UPDRS Part III	23.2 (2.1)	25.2 (1.8)	1.9 (-1.7, 5.5)	23.5 (2.0)	25.5 (1.6)	2.0 (-1.3, 5.3)	-0.4 (-4.4, 3.6)	0.85
MDS-UPDRS Part IV	4.4 (0.7)	4.1 (0.6)	-0.3 (-1.5, 0.8)	2.8 (0.8)	3.7 (0.5)	0.9 (-0.2, 2.0)	0.4 (-0.9, 1.7)	0.54
PDQ-39 Summary Index	25.4 (2.1)	21.2 (2.5)	-4.2 (-9.2, 0.8)	24.1 (2.4)	22.0 (2.3)	-2.1 (-6.7, 2.5)	-0.7 (-6.2, 4.8)	0.79
Rome III Constipation Severity Scale	4.4 (0.7)	3.7 (0.6)	-0.8 (-2.0, 0.5)	5.0 (0.9)	3.9 (0.6)	-1.0 (-2.1, 0.1)	-0.2 (-1.6, 1.1)	0.71

Data are mean (SE) or mean (95% CI). *P*-values for secondary outcomes are not adjusted for multiple comparisons and should be interpreted with caution.

Abbreviations: PAS, Parkinson Anxiety Scale; BDI-II, Beck Depression Inventory-II; IDS-C, Inventory of Depressive Symptomatology, Clinician-Rated; QIDS-SR, Quick Inventory of Depressive Symptomatology, Self-Report; MDS-UPDRS, Movement Disorder Society-Unified Parkinson's Disease Rating Scale; PDQ-39, Parkinson's Disease Questionnaire-39.

[1] The within-group change from baseline was significant at $p < 0.001$.

[2] The within-group change from baseline was significant at $p < 0.01$.

[3] The within-group change from baseline was significant at $p < 0.05$.

Supplementary Table 9. Adverse events.

	**Probiotic (n = 30)**	**Placebo (n = 31)**	***p* (Fisher's exact test)**
Common cold	3	0	0.11
COVID-19 infection	2	3	1.0
Increased anxiety	1	1	1.0
Worsening of tremor	3	1	0.35
Nausea	2	0	0.24
Increased dyskinesias	0	1	1.0
Increased frequency, duration, and severity of OFF period symptoms	0	2	0.49
Any gastrointestinal-related AE	8	10	0.78
Diarrhea	0	2	0.49
Bloating	1	3	0.61
Increased flatulence or belching	5	4	0.73
Constipation	1	1	1.0
Abdominal cramping/pain	0	2	0.49
Increased bowel sounds	1	1	1.0
Increased bowel movements/urgency	0	2	0.49
Hematuria	1	0	0.49
Increased fatigue	1	0	0.49
Dysphagia	0	1	1.0
Sciatica	0	1	1.0
Fall	1	1	1.0
Dizziness	1	1	1.0
Generalized itch	0	1	1.0
Number of participants reported AEs deemed possibly, probably or definitely related to the intervention	10	12	0.79
Total number of AEs	41	45	

Note: a few AEs considered not related or unlikely related are not reported. Reporting method is similar to other studies[14,15].

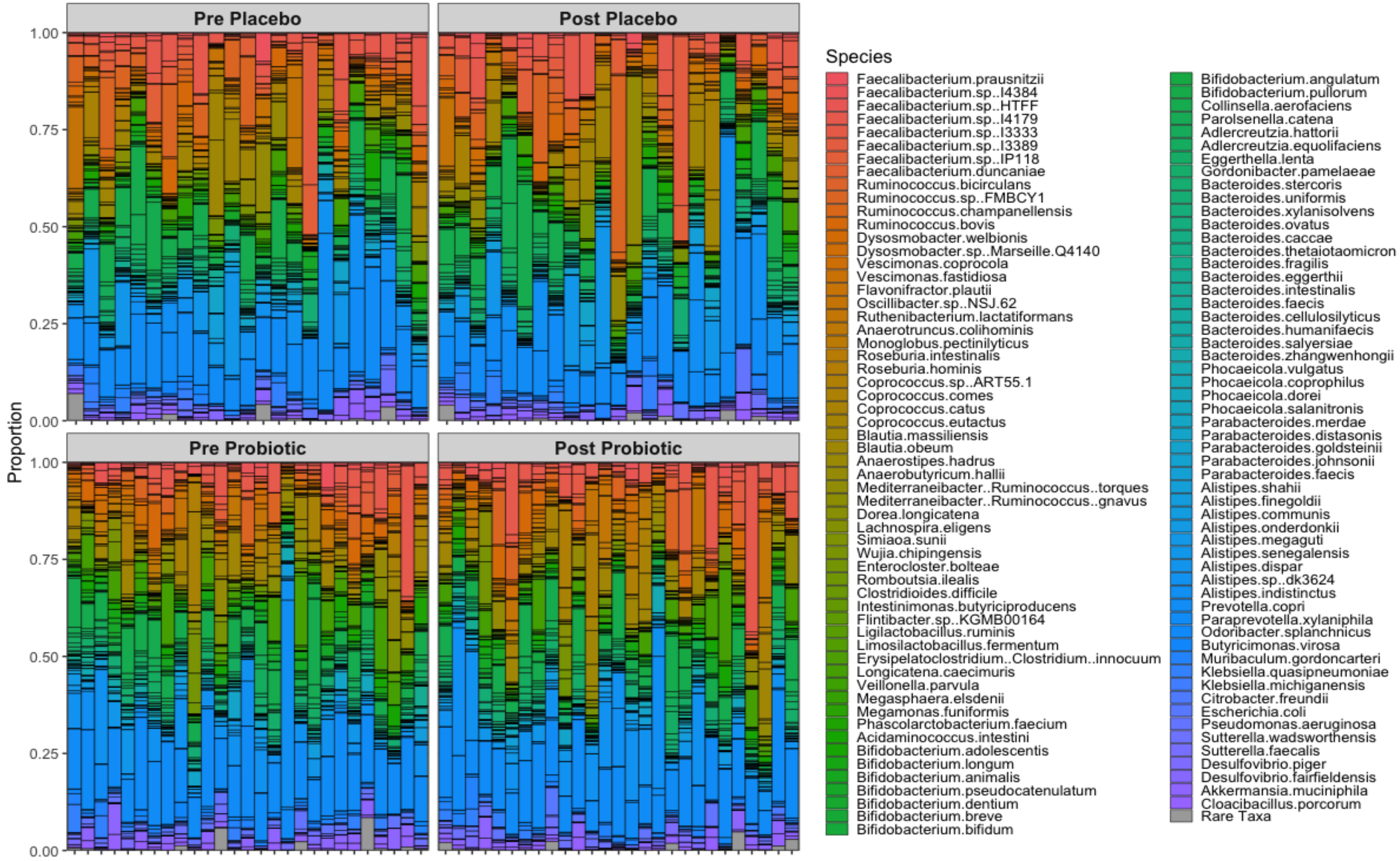

Supplementary Figure 1. Stacked bar plot.

The relative abundances of species-level taxa for each participant are shown. Species that were never detected at a 1% relative abundance or higher are aggregated and defined as rare taxa for the purposes of the stacked bar plots.

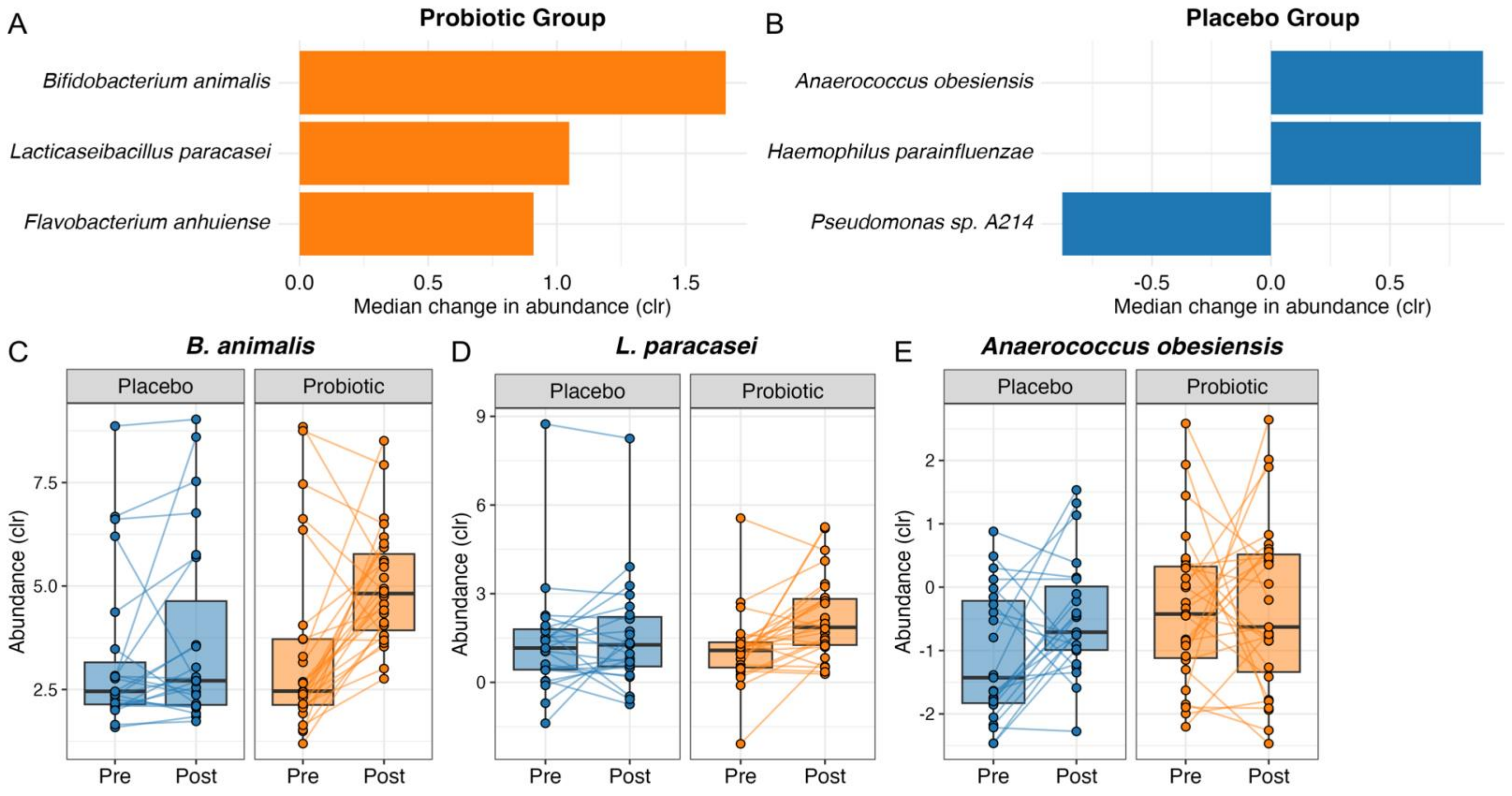

Supplementary Figure 2. Changes in the microbiota following the 12-week intervention.

Top three species that showed the greatest changes in clr-transformed abundance in the probiotic (A) and placebo (B) groups. A negative value indicates reduced abundance; a positive value indicates increased abundance. Abundances of *B. animalis* (C) and *L. paracasei* (D) were increased in the probiotic group, while the relative abundance of *A. obesiensis* was increased in the placebo group, although these increases were not statistically significant after FDR correction. Box plots depict the medians and interquartile ranges; lines connect individual pre- and post-intervention values. To interpret panels (A) and (B) using *B. bifidum* as an example: the median change in clr-transformed abundance corresponds to an approximately 5.23-fold increase ($\exp^{1.655}$) relative to the geometric mean abundance of all taxa following probiotic supplementation.

References

1. Csizmadi, I. *et al.* Using national dietary intake data to evaluate and adapt the US Diet History Questionnaire: The stepwise tailoring of an FFQ for Canadian use. *Public Health Nutr.* **19**, 3247–3255 (2016).
2. Washburn, R. A., Zhu, W., McAuley, E., Frogley, M. & Figoni, S. F. The Physical Activity Scale for Individuals with Physical Disabilities: Development and evaluation. *Arch. Phys. Med. Rehabil.* **83**, 193–200 (2002).
3. Leentjens, A. F. G. *et al.* The Parkinson Anxiety Scale (PAS): Development and validation of a new anxiety scale. *Mov. Disord.* **29**, 1035–1043 (2014).
4. Sheehan, D. V. *et al.* The Mini-International Neuropsychiatric Interview (M.I.N.I.): The development and validation of a structured diagnostic psychiatric interview for DSM-IV and ICD-10. *J. Clin. Psychiatry* **59**, 22–33 (1998).
5. Beck, A. T., Steer, R. A. & Brown, G. K. *Manual for the Beck Depression Inventory-II.* (Psychological Corporation, San Antonio, TX, 1996).
6. Trivedi, M. H. *et al.* The Inventory of Depressive Symptomatology, Clinician Rating (IDS-C) and Self-Report (IDS-SR), and the Quick Inventory of Depressive Symptomatology, Clinician Rating (QIDS-C) and Self-Report (QIDS-SR) in public sector patients with mood disorders: A psychometric evaluation. *Psychol. Med.* **34**, 73–82 (2004).
7. Krupp, L. B., Larocca, N. G., Muir Nash, J. & Steinberg, A. D. The Fatigue Severity Scale: Application to patients with multiple sclerosis and systemic lupus erythematosus. *Arch. Neurol.* **46**, 1121–1123 (1989).
8. Starkstein, S. E. *et al.* Reliability, validity, and clinical correlates of apathy in Parkinson's disease. *J. Neuropsychiatry Clin. Neurosci.* **4**, 134–139 (1992).
9. Nasreddine, Z. S. *et al.* The Montreal Cognitive Assessment, MoCA: A brief screening tool for mild cognitive impairment. *J. Am. Geriatr. Soc.* **53**, 695–699 (2005).
10. Goetz, C. G. *et al.* Movement Disorder Society-sponsored revision of the Unified Parkinson's Disease Rating Scale (MDS-UPDRS): Scale presentation and clinimetric testing results. *Mov. Disord.* **23**, 2129–2170 (2008).
11. Jenkinson, C., Fitzpatrick, R., Peto, V., Greenhall, R. & Hyman, N. The Parkinson's Disease Questionnaire (PDQ-39): Development and validation of a Parkinson's disease summary index score. *Age Ageing* **26**, 353–357 (1997).
12. Drossman, D. A. The functional gastrointestinal disorders and the Rome III process. *Gastroenterology* **130**, 1377–1390 (2006).
13. Wong, R. K. *et al.* Inability of the Rome III criteria to distinguish functional constipation from constipation-subtype irritable bowel syndrome. *Am. J. Gastroenterol.* **105**, 2228–2234 (2010).
14. Chahwan, B. *et al.* Gut feelings: A randomised, triple-blind, placebo-controlled trial of probiotics for depressive symptoms. *J. Affect. Disord.* **253**, 317–326 (2019).
15. Scheperjans, F. *et al.* Fecal microbiota transplantation for treatment of Parkinson disease: A randomized clinical trial. *JAMA Neurol.* **81**, 925–938 (2024).
